# S128. CORRELATION OF DURATION OF UNTREATED PSYCHOSIS WITH TREATMENT RESPONSE ON THE SYMPTOM DIMENSIONS OF SCHIZOPHRENIA

**DOI:** 10.1093/schbul/sby018.915

**Published:** 2018-04-01

**Authors:** Beatriz Yukie Elias, Daniela Koga Tonsig, Bruno Bertolucci, Cristiano Noto, Daniel Azevedo Cavalcante, Quirino Cordeiro, Rodrigo Bressan, Ary Gadelha

**Affiliations:** 1 Federal University of Sao Paolo; 2 Santa Casa de misericordia de São Paulo

## Abstract

**Background:**

Longer duration of untreated psychosis (DUP) predicts worse response to treatment and functional outcomes in first episode of schizophrenia (FES). Longer DUP also seem to particulary affect the severity of negative symptoms, but most studies enrolled previously medicated patients and did not focus on differential effects on schizophrenia symptomatic dimensions. This study investigates how DUP influences the five dimensions of symptoms of schizophrenia on antipsychotic naïve FEP patients before and after two months of treatment.

**Methods:**

Drug-naïve patients at FES (n = 97) were recruited from the Inpatient Psychiatric Unit of Santa Casa de Misericórdia de São Paulo (Sao Paulo, Brazil), between 2011 and 2016. Subjects were assessed at hospital admission and after two months of follow up. All patients were treated with antipsychotics after the diagnosis was confirmed with the Structured Clinical Interview for DSM-IV (SCID-I). The Positive and Negative Syndrome Scale (PANSS) was administered at baseline and after two months of treatment. The PANSS items were grouped in five factors: positive, negative, disorganized/cognitive, mood/depression and excitement/hostility factors. The factors percentage reduction from baseline after treatment were correlated with the DUP, controlled for sex, age, years of education.

**Results:**

The mean years of education of the sample was 9.2 (± 2.6 SD), mean age was 24.9 (± 7.0 SD), 62.9% were male and 42.7% were unemployed or had stopped their studies because of symptoms. Pearson correlation coefficients of the factors with DUP were: Positive = - 0.311 (p < 0.001); Negative= -0.340 (p < 0.001); Disorganized = -0.188 (p = 0.033); Hostility = -0.201 (p= 0.023); Depression = 0.030 (p = 0.389).

**Discussion:**

Shorter DUP enhanced the early response to treatment in the positive, negative, disorganized and hostility dimensions. In line with the literature, our findings support that reducing the DUP may be one of the few interventions for a more favorable response to treatment on negative symptoms.

